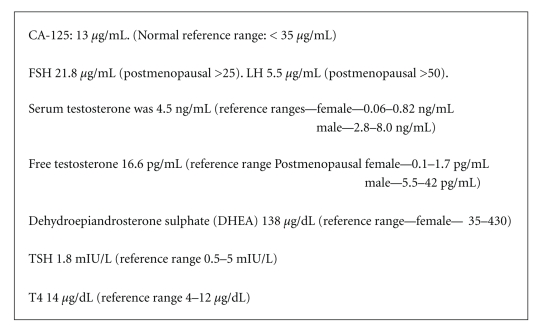# Luteinized Ovarian Thecoma in a Postmenopausal Women Presenting with Virilization

**DOI:** 10.1155/2009/492386

**Published:** 2009-02-25

**Authors:** Athula Kaluarachchi, Jeevan P. Marasinghe, Thuwan M. Batcha, Preethika Agunawela

**Affiliations:** ^1^Department of Obstetrics and Gynaecology, Faculty of Medicine, University of Colombo, Colombo 00800, Sri Lanka; ^2^Professorial Obstetrics and Gynaecology Unit, De Soysa Hospital for Women, Colombo 00800, Sri Lanka; ^3^Department of Pathology, Faculty of Medicine, University of Colombo, Colombo 00800, Sri Lanka

## Abstract

We report a case of luteinizing thecoma in a 58-year-old postmenopausal woman who presented with progressive androgenic features and hypertension of one year duration. She did not notice a significant change in her body weight or appetite. Her total serum testosterone level was 4.5 ng/mL. Ultrasound scan revealed a normal-sized uterus and a right-sided solid ovarian mass of 5 cm × 5 cm. Left ovary was normal. She had a total abdominal hysterectomy, bilateral salpingo-oophorectomy, and an omentectomy performed. Histological examination confirmed the diagnosis of luteinized thecoma. This case illustrates the necessity to consider the rare possibility of luteinized ovarian thecoma as a cause for virilization in a menopausal woman.

## 1. Introduction

Virilization due to hyperandrogenism caused by a luteinized thecoma in a postmenopausal woman is extremely rare.

## 2. Case Report

A 58-year-old postmenopausal woman of two living children presented to the university gynecological unit, National Hospital of Sri Lanka, Colombo, complaining of progressive hair loss and male-type hair distribution of one year duration. These symptoms were accompanied by lower abdominal pain for the last four months duration. She did not notice a significant change in her body weight or appetite. There was no postmenopausal bleeding or vaginal discharge.

She was diagnosed to have hypertension for the last one and half year duration and was on regular treatment with a good control of blood pressure. She had undergone hemithyroidectomy at the age of 44 years due to a cold nodule. Histology had revealed a follicular adenoma and she was treated with L-thyroxine 50 *μ*g daily.

**Figure 1:**
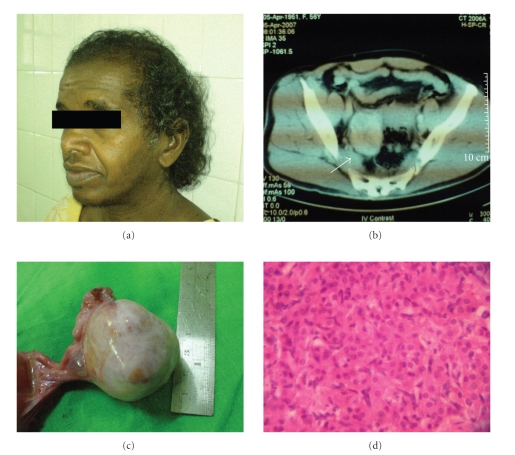
(a) On examination, she had alopecia, frontal balding, and hirsuitism giving a score of 14 from modified Ferriman and Gallwey scoring system. (b) Computed tomography of the abdomen and pelvis revealed a well-defined solid tumor in right adnexal region (arrow). (c) Laparotomy revealed a solid ovarian tumor of 5 cm with an intact capsule in the right ovary. (d) Haematoxyline and eosine-stained sections (magnification 10 × 100) revealed cell nets comprising uniform cells with round bland nuclei and eosinophilic cytoplasm. They were surrounded by a proliferation of elongated spindle cells.

On examination, she had alopecia, frontal balding, and hirsuitism involving the face, chin, upper back, chest, upper and the lower abdomen giving a score of 14 from modified Ferriman and Gallwey scoring system (Figure 1(a)).

She had clitoromegally. Her respiratory and cardiovascular systems were normal. Pelvic examination revealed a solid right ovarian mass of 5 cm size and it was confirmed by pelvic ultrasound scan. It was a solid mass with an intact capsule. The uterus and the other ovary were normal and there was no free fluid. Liver and the other abdominal organs were normal. Computed tomography (CT-scan) of the abdomen and pelvis revealed a well-defined solid tumor of 5.5 × 3 × 6 cm in right adnexal region (Figure 1(b)). Other pelvic structures and the suprarenal glands were normal. Her investigation results were as in Figure 2.

Laparotomy revealed a solid ovarian tumor of 5 cm with an intact capsule in the right ovary. The left ovary and the uterus were normal (Figure 1(c)). There was no free fluid. There were no intraperitoneal deposits. Total abdominal hysterectomy and bilateral salpingo-oophorectomy along with infracolic omentectomy were performed. Patient made an uneventful recovery and was discharged on the fourth postoperative day.

Histological examination of sections from the right ovarian mass stained with haematoxyline and eosine revealed cell nets comprising uniform cells with round bland nuclei and eosinophilic cytoplasm. Mitotic figures were sparse. They were surrounded by a proliferation of elongated spindle cells with bland nuclei (Figure 1(d)). A histological diagnosis of luteinized thecoma was made. The capsule of the mass was intact. The other ovary, uterus, cervix, and the omentum were normal. The endometrium was nonreactive with atrophic changes.

The patient was reviewed two months after the surgery. Hirsuitism was still there, but there was regression of virilism. Her serum testosterone was 0.09 ng/mL (reference ranges—female—0.06–0.82 ng/mL) when she was reviewed ten months after surgery and there was a significant decrease in both hirsuitism and virilism.

## 3. Discussion

Hyperandrogenism is one of the most common endocrine disorders in women accounting for large number of visits to the general practitioners and the gynecologists. Polycystic ovary syndrome accounts for majority of them in the reproductive aged women. Virilization due to hyperandrogenism is rare in postmenopausal women. Hyperandrogenism due to a luteinized thecoma in a postmenopausal woman is extremely rare. Review of the indexed literature (MEDLINE 1966–2008, English language; search terms: luteinized thecoma, Postmenopausal) revealed only a handful of cases of luteinized thecoma in a postmenopausal patient who had presented with virilization and alopecia [1]. There were few other reported cases of ovarian thecoma in postmenopausal woman, where the presenting symptoms were androgenic manifestations, bleeding, and abdominal pain [2, 3]. The histology of these patients did not reveal luteinization.

Ovarian thecoma is a rare hormonally active tumor of stromal cell origin and represents less than 1% of all ovarian tumors. It occurs most often in perimenopausal and postmenopausal women [4]. Seventy percent of thecomas secrete oestrogens and usually presents with postmenopausal bleeding and with endometrial hyperplasia or malgnancy [5]. Nevertheless, androgens and exceptionally progesterone or corticosteroids are occasionally produced [6]. When the tumor contains luteinized cells, which is observed in 10% of thecomas [6], they secrete androgens and lead to virilization which was observed in our patient. Typical picture of androgen secretion is oligomenorrhoea, defeminization, and progressive virilization (acne, hirsuitism, temporal balding, enlargement of clitoris, deepening of the voice, and muscular development). Our patient developed complete change of the external appearance within a short period of time. This change created a socially embarrassing situation which limited her social interaction as well as physical performances.

The diagnosis of a virilizing ovarian tumor is most often complicated by the findings of clinical features suggestive of Cushing's syndrome. However, in our patient an adrenal tumor and Cushing's syndrome were excluded by ultrasound scan, CT, of the abdomen and by biochemical tests.

Elevation of serum testosterone level above 2 ng/mL has been proposed as a significant level which indicates the presence of an androgen secreting tumor. Testosterone levels in polycystic ovarian disease and stromal hyperthecosis are often below this level. Serum testosterone level of 4.5 ng/mL in our patient was strongly indicative of the presence of an androgen secreting ovarian tumor [7]. Size of most ovarian thecomas ranges from 5–10 cm and is bilateral in 3% of cases [8]. Our patient had a unilateral tumor measuring about 5 cm which was detected on ultrasound and CT-scan.

Surgical intervention is generally adopted as the primary mode of treatment. Owing to the postmenopausal status of our patient, it was recommended to proceed with total abdominal hysterectomy and bilateral salpingo-oophorectomy as therapy for a presumed ovarian malignancy. However, histology of the affected ovary did not reveal any possibility of malignancy. Serum testosterone level returned to normal after surgery and the patient was happy and contended when she was reviewed ten months after surgery since there was significant improvement in her clinical status. This case illustrates the necessity to consider the rare possibility of luteinized ovarian thecoma as a cause for virilization in a menopausal woman.

## Figures and Tables

**Figure 2:**